# Photoperiod and Melatonin Supplementation: Variable Effects on the Quality of Chilled Dog Semen

**DOI:** 10.3389/fvets.2022.956630

**Published:** 2022-07-12

**Authors:** Olga Mitjana, Raquel Ausejo, Noelia Mendoza, Joaquin Miguel, Maria Teresa Tejedor, Ana Maria Garrido, Maria Victoria Falceto

**Affiliations:** ^1^Agroalimentary Institute of Aragon-IA2, Department of Animal Pathology, Universidad de Zaragoza-CITA, Zaragoza, Spain; ^2^Department of Biotechnology R&D, Magapor S.L., Ejea de los Caballeros, Spain; ^3^Department of Animal Pathology, Universidad de Zaragoza-CITA, Zaragoza, Spain; ^4^Department of Anatomy, Embryology and Animal Genetics, CiberCV, Universidad de Zaragoza, Zaragoza, Spain

**Keywords:** photoperiod, melatonin supplementation, antioxidant, chilled dog semen, quality

## Abstract

The addition of melatonin in seminal extenders due to its antioxidant properties and its beneficial role in sperm preservation has been previously described, especially in seasonal species. The aim of this study was to study a potential seasonal effect based on photoperiod duration when adding a physiological concentration of melatonin in the canine ejaculate. A total of 24 ejaculates were obtained from 10 healthy dogs during the increasing photoperiod (from December 21 to June 21), whereas 12 ejaculates were collected from five healthy individuals during the decreasing photoperiod (from June 22 to December 20). Each ejaculate was separated into two aliquots, and one of them remained as a control, whereas melatonin (100 pM) was added to the other one (C and M treatment groups, respectively). Diluted semen was refrigerated at 5°C. On days 0, 1, 2, 3, and 6, sperm motility analyses were performed using a CASA system and hypoosmotic swelling test (HOST), osmotic resistance test (ORT), and flow cytometry analysis. No effect of melatonin on motility was detected in either photoperiod. Negative effects of melatonin were found for acrosomal defects, apoptosis, and viability in the decreasing photoperiod. The addition of melatonin to sperm in the decreasing photoperiod could create such a high level that it would cause the described negative effects. We found a beneficial effect of melatonin in the increasing photoperiod on acrosomal defects and apoptosis during 0–6 days. Melatonin treatment also increased viability in the short term (days 1 and 2) for both photoperiods. Also, melatonin can provide certain beneficial effects on mitochondrial activity in the medium term (days 2 and 3) in the decreasing photoperiod.

## Introduction

Melatonin (N-acetyl-5-methoxytryptamine) is a lipophilic molecule synthesized from the tryptophan amino acid and detected in vertebrates, invertebrates, bacteria, unicellular organisms, and even plants (1). This endogenous neurohormone is mainly produced by the pineal gland. As well known, it regulates the circadian clock, immunological enhancement, sexual behavior, and seasonal reproduction in animals (2, 3), being that its secretion is regulated by the light/dark cycle (4).

However, melatonin is also synthesized by other organs, such as the cerebellum, retina, skin, gastrointestinal cells, Harder's gland, thymus, mononuclear peripheral cells, placenta, ovary, testes, bone marrow, liver, hippocampus, and platelets (1, 5). Therefore, it accomplishes other biological functions (immunomodulation, anti-inflammatory, and antioxidant activities) (6, 7). Melatonin shows antioxidant activity and free radical scavenging ability (8) and stimulates the activity of antioxidant enzymes (superoxide dismutase, glutathione peroxidase, and catalase) (7, 9).

Melatonin and melatonin receptors (MT1 and MT2) are universally distributed in the reproductive systems of mammals; it has been suggested that these receptors in spermatozoa may be related to protection against oxidative damage rather than seasonal reproduction (10). Hence, melatonin has been widely used as a potent antioxidant for semen cryopreservation in mammals (11).

In dogs, both freezing–thawing and long-term cold storage harm spermatozoa (12), and oxidative stress (excessive accumulation of the unscavenged free oxygen and nitrogen radicals) is the main cause of this damage (13). The protective effect of melatonin is species-specific, and even individual factors may play a role (14). On the contrary, the negative effect of excess melatonin concentration on spermatozoa has been described (15). Recently, several studies have observed the effect of melatonin supplementation on canine semen quality using several melatonin concentrations, e.g., 0.0005–0.0035 mol/L (16) and 0.5–2 mM (17). However, no reference to potential seasonal effects was made. The mean melatonin concentration in canine ejaculate was 6.06 ± 2.28 pg/ml, but with large individual variation independent of breed and age, being that it has a maximum value of 29 pg/ml; these data came from seven dogs whose individual semen samples were collected between March and June (10). Therefore, the objective of this study was to study a new factor in the variability of melatonin effects on canine semen quality: a potential seasonal effect based on photoperiod duration with a melatonin dose close to the physiological concentration of the ejaculate of a dog.

## Materials and Methods

### Sperm Collection

A total of 24 ejaculates were obtained from 10 healthy dogs (two ejaculates from each of the eight dogs and four ejaculates from each of the two dogs), during the increasing photoperiod (from December 21 to June 21), whereas 12 ejaculates were collected from five healthy individuals during the decreasing photoperiod (from June 22 to December 20; two ejaculates from each of the four dogs and four ejaculates from one dog). All of them were living in Zaragoza (Northeastern Spain: 41°39'21.8” N 0°52.64' W). The dogs belonged to private owners, including seven purebreds (Beagle: 1; French Bullgdog: 2; Border Collie: 2; Labrador Retriever: 1; and Golden Retriever:1) and eight mongrels, with a mean age of 2.7 ± 3.5 years.

Two dogs employed to collect semen during the decreasing photoperiod were from the same group of ten dogs employed during the increasing photoperiod (two ejaculates from one of them and four ejaculates from the other one for both photoperiods). Ideally, a single set of dogs should have been studied at both photoperiods; a paired test should have been applied, where each individual would act as its own control, thus increasing the test power. Unfortunately, only two dogs studied in increasing photoperiod were available in decreasing photoperiod; therefore, both experiences were treated as independent.

Ejaculate was obtained by manual masturbation, separating the three fractions. Only the second rich fraction was used for the experiment, which was diluted with the non-commercial diluent Tris-fructose-egg yolk extender (18). The inclusion criterion was that the ejaculate had a total motility ≥70% with a percentage of abnormal forms ≤ 30%.

All procedures in these experiments were conducted consistently with the precepts of animal welfare and approved by the Committee of Ethics in Animal Experimentation of Universidad de Zaragoza (Protocol No. PI09/21NE). Written informed consent was obtained from the owners for the participation of their animals in this study.

### Sperm Sample Treatments

On the extraction day (day 0), an initial analysis of semen quality was carried out, based on the parameters described below. Then, each ejaculate was separated into two aliquots: One of them remained as the control, while melatonin was added to the other one (C and M treatment groups, respectively).

Semen doses were supplemented with 100 pM (23.11 picograms/ml) of melatonin (Sigma-Aldrich) and then preserved for 7 days at 5°C. Another aliquot was maintained in the same conditions with no added melatonin (control sample). To evaluate sperm quality, several parameters, including viability, mitochondrial potential, acrosome integrity, and early apoptosis by flow cytometry and motility by the CASA system, were analyzed at 1, 2, 3, and 6 days.

### Sperm Motility Analysis

Motility kinematics parameters underwent computer-assisted measurement using a CASA system (ISAS 1.0.4; Proiser SL, Valencia, Spain) with a video camera mounted on a microscope (Nikon Eclipse 50i; Nikon, Tokyo, Japan) equipped with a 10 × negative-phase contrast lens and a 10 × projection ocular. Aliquots of 1 ml were warmed up for 5 min at 37°C. Samples (6 μl) were placed between prewarmed slides and coverslips and maintained at 37°C during analysis by a heated slide holder. From a single field, 10 consecutive digitalized images were analyzed.

The following motion characteristics were compared between the control and treated groups: the percentage of total motile (TM) and progressive motile (PM) spermatozoa; curvilinear velocity (VCL), the mean path velocity of the sperm head along its actual trajectory (μm/s); linear velocity (VSL), the mean path velocity of the sperm head along a straight line from its first to last position (μm/s); mean velocity (VAP), the mean velocity of the sperm head along its average trajectory (μm/s); linearity coefficient (LIN: VSL/VCL) × 100 (%); straightness coefficient (STR: VSL/VAP) × 100 (%); and wobble coefficient (WOB: VAP/VCL) × 100 (%).

### Sperm Analysis

Sperm morphology, short hypoosmotic swelling test (sHOST), and short osmotic resistance test (sORT) were assessed for the evaluation of the functional integrity of the sperm membrane and acrosome, which reflects the viability of spermatozoa (19). The sHOST test was performed according to the original test (20) but using a lower osmotic pressure (75 vs. 150 mOsm/kg) and a shorter period of time (15 vs. 120 min), as previously described (21). Eosin-nigrosin stain under a light field microscope at 1,000 × magnification was used to determine the percentage of viable spermatozoa (with curled tails) and the percentage of spermatozoa with intact acrosome. The semen concentration was calculated using a Bürker chamber at a 1/100 dilution (sperm/0.3% formol saline solution).

### Flow Cytometry Analysis

All of the measurements were performed on a BD Accuri™ C6 (Becton, Dickinson and Company, Madrid, Spain) with BD software. At a minimum, 40, 000 events were counted in all of the experiments. The sperm population was gated for further analysis based on its specific forward (FS) and side scatter (SS) properties; other non-sperm events were excluded.

### Viability/Acrosome Integrity

A double-staining technique with propidium iodide, a nuclear dye that penetrates inside the damaged plasmatic membrane in nonviable spermatozoa, and FITC-PNA staining that penetrates when spermatozoa have an acrosome reaction were performed. A volume of 5 μl of each stain (FITC; 1 μg/ml; Sigma) and propidium iodide (PI; 0.75 mM; Sigma) was added to 300 μl of sperm samples (4 × 10^7^ cells per milliliter). Samples were incubated at 37°C in darkness for 15 min. The argon laser and filters of 525 and 675 nm were used to avoid overlapping.

### Early Apoptosis/Mitochondrial Activity

A double-staining technique was performed. YoPro [1 mM in dimethyl sulfoxide (DMSO); Invitrogen], a DNA dye, was used as an apoptotic marker because it only permeates into cells that are beginning to undergo apoptosis, and MitoTracker (10 μM in DMSO, Invitrogen) was used to evaluate mitochondrial membrane potential (ΔΨm). A volume of 2 μl of each dye was added to 300 μl (4 × 10^7^) of spermatozoa, and samples were incubated at 37°C in darkness for 30 min. YoPro emissions were collected with a 525-nm filter and MitoT with a 675-nm filter to avoid spectral overlap.

### Statistical Analysis

The statistical analysis was carried out using IBM SPSS Statistics 26.0 software (IBM Corp., Armonk, NY, USA). The considered semen parameters were submitted to the Shapiro–Wilks test to assess their normal distribution. The following variables showed an approximately normal distribution: TM, PM, VAP, VCL, VSL, STR, LIN, and WOB. Their results were expressed as mean ± SD. In data whose distribution skewed to the right, a logarithmic transformation was applied (% acrosomal defects and % ORT); results were resumed as geometric mean and geometric standard deviation (dispersion factor, DF). Finally, for data dealing with probabilities, percents, and proportions close to either 0 or 1, an arcsine transformation was applied (% apoptosis, % endosmosis, % viability, and % mitochondrial activity); these results were shown as back-transformed mean (modified mean) and modified 1 SD interval.

Results from photoperiods, treatments, and days are shown in Tables 1, 2. On day 0, no significant differences were found between both photoperiods for any parameter (*P* > 0.050).

**Table 1:** Results from CASA motility parameters from semen samples collected in increasing photoperiod during a cold storage period of 6 days.

**Parameter**	**N/group**	**Group**	**Day 0**	**Day 1**	**Day 2**	**Day 3**	**Day 6**
TM (%)	19	C	88.3, 9.66^a^	89.3, 7.37^1a^	85.9, 9.63^1a^	85.9, 9.47^1a^	85.0, 13.25^1a^
		M		85.8, 13.51^1a^	86.1, 10.39^1a^	85.1, 13.43^1a^	84.7, 12.80^1a^
PM (%)	24	C	57.8, 18.80^a^	47.9, 21.28^1a^	53.0, 19.18^1a^	55.3, 20.95^1a^	49.3, 28.36^1a^
		M		51.6, 24.00^1a^	54.9, 20.39^1a^	57.7, 20.96^1a^	48.1, 21.63^1a^
VAP (μm/s)	24	C	46.1, 23.46^a^	49.2, 24.77^1a^	50.0, 23.94^1a^	46.4, 21.71^1a^	42.2, 22.95^1a^
		M		48.7, 22.13^1a^	48.7, 23.75^1a^	43.8, 21.28^1a^	36.9, 16.04^1a^
VCL (μm/s)	24	C	65.4, 33.11^a^	68.9, 36.51^1a^	67.5, 34.81^1a^	65.6, 32.68^1a^	58.6, 34.07^1a^
		M		65.6, 30.12^1a^	65.9, 32.86^1a^	59.3, 28.02^1a^	50.3, 24.51^1a^
VSL (μm/s)	24	C	30.5, 18.05^a^	34.0, 17.17^1a^	34.8, 17.90^1a^	32.4, 16.11^1a^	29.91, 16.23^1a^
		M		35.1, 17.05^1a^	34.9, 19.15^1a^	32.2, 17.81^1a^	27.0, 12.28^1a^
STR (VSL/VAP, %)	23	C	65.4, 10.14^a^	71.3, 11.75^1b^	70.2, 9.21^1ab^	70.4, 8.77^1ab^	71.4, 8.70^1b^
		M		71.8, 9.70^1b^	71.0, 12.13^1ab^	72.4, 11.72^1ab^	73.1, 9.76^1b^
LIN (VSL/VCL, %)	23	C	47.9, 13.24^a^	53.6, 14.64^1b^	53.3, 12.00^1ab^	51.7, 10.67^1ab^	53.6, 11.52^1ab^
		M		54.5, 11.76^1b^	54.5, 14.96^1ab^	55.2, 15.07^1ab^	55.9, 12.49^1ab^
WOB (VAP/VCL, %)	23	C	72.0, 9.02^a^	73.9, 9.27^1a^	74.3, 6.64^1a^	72.9, 7.36^1a^	74.3, 8.20^1a^
		M		75.1, 6.98^1a^	75.4, 8.57^1a^	75.0, 9.31^1a^	75.5, 8.26^1a^

*N/group, sample size (aliquots) per group. Measures for day 0 were carried out before division in two aliquots for C and M groups; therefore, there is only one value for day 0, and the sample size equals N/group. Data are mean ± standard deviation. For each variable, the different numbers and lowercase letters in superscripts indicate a statistically significant difference (p < 0.05) within (C group vs. M group) and between columns (between days), respectively*.

**Table 2:** Results of semen analysis of osmotic resistance test (ORT), endosmosis (HOST), and flow cytometry parameters from semen samples collected in increasing photoperiod during a cold storage period of 6 days.

**Parameter**	**N/group**	**Group**	**Day 0**	**Day 1**	**Day 2**	**Day 3**	**Day 6**
Osmotic resistance test (ORT, %)^A^	20	C	91.5 (1.05)^a^	91.8 (1.03)^1a^	88.2 (1.06)^1a^	81.4 (1.08)^1b^	74.1 (1.15)^1b^
		M		90.2 (1.05)^1a^	85.9 (1.07)^1ab^	83.5 (1.04)^1b^	67.5 (1.14)^2c^
Endosmosis (HOST, %)^B^	24	C	92.6 (86.67 ÷ 96.92)^a^	90.6 (83.04 ÷ 96.12)^1a^	86.9 (78.50 ÷ 93.52)^1b^	82.1 (72.71 ÷ 89.82)^1c^	68.2 (55.32 ÷79.86)^1d^
		M		92.0 (87.01 ÷ 95.84)^1a^	87.3 (80.85 ÷ 92.66)^1b^	83.5 (74.56 ÷ 90.84)^1c^	66.2 (51.09 ÷ 79.83)^1d^
Acrosomal defects(%)^A^	20	C	3.7 (1.73)^a^	5.6 (1.58)^1b^	10.3 (1.45)^1c^	13.1 (1.39)^1d^	17.5 (1.51)^1e^
		M		4.9 (1.64)^2b^	8.5 (1.42)^2c^	11.3 (1.35)^2d^	15.1 (1.40)^2e^
Apoptosis (%)^B^	20	C	4.9 (2.83 ÷ 7.53)^a^	7.1 (4.87 ÷ 9.84)^1a^	9.9 (7.84 ÷ 12.29)^1b^	13.1 (10.77 ÷ 15.71)^1c^	18.2 (15.75 ÷ 20.86)^1d^
		M		6.4 (4.56 ÷ 8.49)^2a^	9.3 (7.75 ÷ 11.02)^2b^	12.4 (10.61 ÷ 14.35)^2c^	17.4 (15.38 ÷ 19.55)^2d^
Viability (%)^B^	20	C	95.6 (90.15 ÷ 98.95)^a^	92.5 (87.59 ÷ 96.23)^1b^	87.7 (81.21 ÷ 92.91)^1c^	83.7 (77.64 ÷ 89.03)^1d^	80.2 (74.69 ÷ 85.15)^1e^
		M		93.9 (89.52 ÷ 97.11)^2a^	89.3 (83.15 ÷ 94.20)^2b^	83.7 (77.36 ÷ 89.11)^1c^	80.2 (75.18 ÷ 84.84)^1d^
Mitochondrial activity (%)^B^	15	C	95.2 (90.62 ÷ 98.33)^a^	95.0 (90.99 ÷ 97.94)^1a^	91.5 (88.51 ÷ 94.16)^1b^	85.7 (81.57 ÷ 89.33)^1c^	82.2 (78.23 ÷ 85.92)^1d^
		M		94.7 (90.61 ÷ 97.72)^1a^	90.0 (86.16 ÷93.27)^1b^	86.12 (82.92 ÷ 89.04)^1c^	82.1 (78.55 ÷ 85.43)^1d^

*N/group, sample size (aliquots) per group. Measures for day 0 were carried out before division in two aliquots for C and M groups; therefore, there is only one value for day 0, and the sample size equals N/group. A, Geometric mean (deviation factor); B, Modified mean (limits of 1 SD area). For each variable, the different numbers and lowercase letters in superscripts indicate a statistically significant difference (p < 0.05) within (C group vs. M group) and between columns (between days), respectively*.

For any parameter, data on day 0 from both photoperiods were compared by means of a one-way ANOVA. A two-way repeated-measures ANOVA was run to determine the effect of the melatonin treatment overtime on the considered semen parameters. When a statistically significant interaction between treatment and day was detected on these parameters, simple main effects were run. If no statistically significant interaction was found, the main effects for both treatment and day were interpreted, including multiple pairwise comparisons with Bonferroni's correction when adequate. For all these analyses, *P*-values < 0.050 were considered statistically significant.

## Results

Results from photoperiods, treatments, and days are shown in Tables 1–**4**. As mentioned earlier, data on day 0 were estimated from ejaculates before separating them into aliquots (C and M groups). On day 0, no significant differences were found between both photoperiods for any parameter (*P* > 0.050).

Tables 1, 2 show the results from the studied semen parameters in the increasing photoperiod. Sample size varied from the initial situation (*N* = 24) depending on the availability of valid data; as time went by, some aliquots did not present live semen due to conservation problems, and no data were available from late days. Only the aliquots with valid data on all days and for both groups can be considered by the ANOVA.

No statistically significant interaction treatment x day (*P* > 0.050) was detected for most of the studied semen parameters (TM, PM, VAP, VCL, VSL, STR, LIN, WOB, acrosomal defects, endosmosis, and mitochondrial activity). Therefore, both treatments did not have different effects on these parameters over time. The main effect of treatment only showed a significant difference for acrosomal defects (*F* = 11.868: *P* = 0.003), with lower values in the M group regardless of time. Significant differences among days were only detected for two CASA motility parameters, namely, STR (*F* = 4.681; *P* = 0.002) and LIN (*F* = 2.928; *P* = 0.025), as well as for acrosomal defects ( *F*= 196.962; *P* < 0.001), endosmosis (HOST; *F* = 57.558; *P* < 0.001), and mitochondrial activity (*F* = 69.147; *P* < 0.001).

There was statistically significant interaction treatment × day on the ORT (*F* = 4.135; *P* = 0.027), apoptosis (*F* = 4.864; *P* = 0.024), and viability (*F* = 4.760; *P* = 0.028). Therefore, for these variables, treatments acted differently over time. For ORT, the M group only differed significantly from the C group on day 6 (*P* = 0.020); the ORT value was lower for the M group. Values from the M group were always significantly lower than the control ones for apoptosis (*P* <0.010). Only on days 1 and 2 did the M group showed significantly higher viability than the C group (*P* < 0.010). On the contrary, ORT and viability decreased significantly over time in both treatment groups (*P* < 0.001).

Tables 3, 4 show the results from the studied semen parameters in the decreasing photoperiod. Also, the sample size varied from the initial situation (*N* = 12) due to the same reasons as in the increasing photoperiod.

**Table 3:** Results from CASA motility parameters from semen samples collected in decreasing photoperiod during a cold storage period of 6 days.

**Parameter**	**N/Group**	**Group**	**Day 0**	**Day 1**	**Day 2**	**Day 3**	**Day 6**
TM (%)	10	C	86.0, 19.40	86.2, 15.65^1^	89.1, 11.32^1^	87.6, 9.17^1^	71.5, 31.39^1^
		M		89.7, 9.74^1^	95.0, 3.82^1^	82.2, 25.88^1^	67.9, 33.11^1^
PM (%)	10	C	61.9, 27.80^a^	52.2, 20.28^1a^	61.6, 23.07^1a^	59.9, 21.81^1a^	47.6, 28.21^1a^
		M		64.1, 20.73^1a^	70.9, 16.72^1a^	52.5, 25.09^1a^	47.4, 27.03^1a^
VAP (μm/s)	10	C	58.7, 31.49^a^	54.4, 28.33^1a^	57.1, 27.67^1a^	57.5, 28.25^1a^	51.6, 34.60^1a^
		M		59.5, 24.40^1a^	69.8, 31.11^1a^	51.2, 27.97^1a^	50.4, 31.50^1a^
VCL (μm/s)	10	C	76.1, 41.26^a^	75.5, 42.13^1a^	76.6, 37.21^1a^	74.0, 33.76^1a^	67.1, 43.81^1a^
		M		78.9, 34.63^1a^	91.6, 39.40^1a^	69.9, 36.17^1a^	64.9, 39.83^1a^
VSL (μm/s)	10	C	42.6, 23.12^a^	39.3, 23.25^1a^	43.8, 23.72^1a^	43.8, 24.08^1a^	41.7, 30.45^1a^
		M		45.5, 21.09^1a^	52.9, 27.36^1a^	37.7, 23.34^1a^	42.2, 29.14^1a^
STR (VSL/VAP, %)	10	C	72.3, 6.78^a^	71.6, 16.30^1a^	75.7, 11.90^1a^	74.4, 13.23^1a^	68.8, 26.43^1a^
		M		75.4, 10.67^1a^	72.8, 10.87^1a^	70.3, 15.60^1a^	71.5, 27.86^1a^
LIN (VSL/VCL, %)	10	C	56.1, 7.50^a^	54.4, 19.40^1a^	57.4, 13.67^1a^	58.3, 15.83^1a^	52.5, 21.55^1a^
		M		58.3, 13.08^1a^	55.7, 13.07^1a^	52.0, 18.28^1a^	55.7, 24.50^1a^
WOB (VAP/VCL, %)	10	C	77.2, 3.55^a^	73.9, 10.62^1a^	74.9, 7.50^1a^	77.1, 10.54^1a^	68.2, 24.43^1a^
		M		76.4, 7.87^1a^	75.6, 6.85^1a^	72.0, 10.56^1a^	69.1, 25.81^1a^

*N/group, sample size (aliquots) per group. Measures for day 0 were carried out before division in two sub-aliquots for C and M groups; therefore, there is only one value for day 0, and the sample size equals N/group. Data are mean ± standard deviation. For each variable, the different numbers and lowercase letters in superscripts indicate a statistically significant difference (p < 0.05) within (C group vs. M group) and between columns (between days), respectively. For TM, no superscript was included related to days (i.e., multiple pairwise comparisons failed to find significant differences between days)*.

**Table 4:** Results of semen analysis of osmotic resistance test (ORT), endosmosis (HOST), and flow cytometry parameters from semen samples collected in decreasing photoperiod during a cold storage period of 6 days.

**Parameter**	**N/Group**	**Group**	**Day 0**	**Day 1**	**Day 2**	**Day 3**	**Day 6**
Osmotic resistance test (ORT, %)^A^	11	C	90.7 (1.04)^a^	85.6 (1.05)^1bce^	82.3 (1.07)^1cde^	78.7 (1.05)^1de^	65.2 (1.31)^1e^
		M		85.0 (1.03)^1bce^	79.6 (1.08)^1cde^	78.3 (1.08)^1de^	65.3 (1.27)^1e^
Endosmosis (HOST, %)^B^	11	C	89.3 (82.61 ÷ 94.51)^ab^	89.4 (82.54 ÷ 94.72)^1a^	87.1 (78.79 ÷ 93.57)^1ab^	84.2 (75.91÷ 91.69)^1ab^	79.7 (66.60 ÷ 90.14)^1b^
		M		87.9 (79.43 ÷ 94.41)^1a^	87.4 (79.28 ÷ 93.79)^1ab^	85.8 (78.58 ÷ 91.82)^1ab^	79.9 (69.16 ÷ 88.81)^1b^
Acrosomal defects (%)^A^	12	C	4.4 (1.35)^a^	6.2 (1.35)^1b^	9.9 (1.17)^1cd^	13.2 (1.60)^1de^	18.8 (1.09)^1e^
		M		6.5 (1.47)^1bc^	8.9 (1.18)^2c^	19.2 (1.10)^2d^	24.3 (1.17)^2e^
Apoptosis (%)^B^	12	C	4.5 (2.65 ÷ 6.86)^a^	9.6 (8.30 ÷ 11.04)^1bc^	9.0 (7.18 ÷ 11.09)^1cd^	11.8 (9.63 ÷ 14.12)^1de^	12.6 (9.99 ÷ 15.47)^1e^
		M		10.3 (7.21 ÷ 13.94)^2bc^	9.3 (7.68 ÷ 11.16)^2cd^	11.3 (8.75 ÷ 14.07)^2de^	15.8 (13.34 ÷ 18.35)^2e^
Viability (%)^B^	12	C	92.3 (87.30 ÷ 96.17)^ab^	91.0 (87.10 ÷ 94.34)^1a^	89.3 (85.79 ÷ 92.37)^1a^	90.5 (87.91 ÷ 92.81)^1a^	87.0 (84.54 ÷ 89.37)^1a^
		M		94.7 (93.17 ÷ 96.15)^2a^	89.3 (87.81 ÷ 90.73)^1bcd^	86.0 (82.74 ÷ 88.97)^2cd^	83.0 (76.64 ÷ 88.53)^2cd^
Mitochondrial activity (%)^B^	12	C	95.8 (92.57 ÷ 98.16)^ab^	94.0 (91.72 ÷ 95.95)^1a^	88.3 (76.01 ÷ 96.63)^1ac^	83.5 (71.52 ÷ 92.75)^1bc^	88.0 (83.51 ÷ 91.95)^1c^
		M		92.9 (90.50 ÷ 95.02)^1ab^	95.9 (92.87 ÷ 98.12)^2b^	87.7 (78.19 ÷ 94.80)^2ac^	87.8 (85.52 ÷ 89.86)^1c^

*N/group, sample size (aliquots) per group. Measures for day 0 were carried out before division in two aliquots for C and M groups; therefore, there is only one value for day 0, and the sample size equals N/group. A, Geometric mean (deviation factor); B, Modified mean (limits of 1 SD area). For each variable, the different numbers and lowercase letters in superscripts indicate a statistically significant difference (p < 0.05) within (C group vs. M group) and between columns (between days), respectively. For apoptosis, the global mean value was higher in the M group (9.9, modified 1 SD area, 5.87 ÷14.91) than in the C group (9.3, modified 1 SD area, 5.91 ÷13.33) (F = 5.982; p = 0.032)*.

No statistically significant interaction treatment × day (*P* > 0.050) was detected for most of the studied semen parameters, although they were not the same parameters as in the increasing photoperiod (TM, PM, VAP, VCL, VSL, STR, LIN, WOB, ORT, apoptosis, and endosmosis). No statistically significant interaction treatment × day (*P* > 0.050) was detected for most of the studied semen parameters. The main effect of treatment only showed a significant difference for apoptosis (*F* = 5.982; *P* = 0.032); the global mean value was higher in the M group (9.9, modified 1 SD area: 5.87 ÷ 14.91) than in the C group (9.3, modified 1 SD area: 5.91 ÷ 13.33). Significant differences among days were only detected for TM (*F* = 2.824; *P* = 0.039), ORT (*F* = 12.652; *P* = 0.003), apoptosis (*F* = 68.468; *P* < 0.001), and HOST (*F* = 5.777; *P* < 0.001). Multiple pairwise comparisons for TM failed to find significant differences between days; therefore, no superscript was included that is related to days for this variable in Table 3.

In the decreasing photoperiod, significant interactions between treatment and day were found on acrosomal defects (*F* = 5.192; *P* = 0.017), viability (*F* = 11.229; *P* < 0.001), and mitochondrial activity (*F* = 9.112; *P* =0.003). Treatments acted differently over time with these variables. On day 2, acrosomal defects were lower for the M group (*P* = 0.019), but on both days 3 and 6, these values were higher for the M group (*P* = 0.019 and *P* < 0.001, respectively); no significant difference was found on day 1 (*P* = 0.621). For the M group, viability was higher than the control on day 1 (*P* = 0.003), had no difference on day 2 (*P* = 0.990), and was lesser on day 3 (*P* = 0.001) and day 6 (*P* = 0.014). Mitochondrial activity was significantly higher for the M group only on day 2 (*P* = 0.008) and day 3 (*P* < 0.001); no other significant differences were found (*P* > 0.050).

## Discussion

The best protective melatonin concentration must be found for each species because its effects on sperm show great inter- and intraspecific variability; in mammals, the best dose ranges from 0.00001 mM to 0.003 mol/L (14, 17). On the contrary, high melatonin doses may reduce sperm motility and viability after cryopreservation, as a consequence of excessive ROS neutralization that inhibits oxidative phosphorylation (15).

Dose-dependent effects seem to be very important in dogs. Doses of 1 mM of melatonin did not affect motility and acrosome integrity (22); even 2 mM doses did not affect motility, plasma membrane integrity, acrosome integrity, capacitation status, and membrane fluidity after freezing–thawing (14). However, 0.002 and 0.0035 mol/L of melatonin conserved canine kinematic parameters when compared with a control group, without added melatonin (1). The addition of 1–2 mM of melatonin could also improve the sperm motility of canine sperm (17). In canine sperm, 0.002 and 0.0035 mol/L of melatonin received good results in the conservation of mitochondrial activity (16). The effect of melatonin on the acrosome in dogs is controversial. Doses of 1 mM of melatonin (22) did not affect acrosome integrity. More recent studies have found that melatonin reduced the percentage of acrosome-damaged sperm in dogs when used in 0.002 and 0.0035 mol/L (16) and even in 1 to 2 mM concentrations (17).

Recently, chilled canine semen can survive until 10–14 days due to modern extenders; however, sperm quality decreases during cold storage (23). In this study, mobility parameters, TM, PM, VAP, VCL, VSL, and WOB, did not vary significantly over time in both photoperiods. The short-day interval considered (0–6 days) could explain these facts. Punctual higher values in certain days for STR (days 1 and 6) and LIN (day 1) in the increasing photoperiod could be occasional. The longevity of canine spermatozoa can be extended up to 14 days of cold storage at 5°C (51). Divar et al. (17) studied the canine sperm motility during long-term storage (9 days) at 5°C, both with and without added melatonin. Melatonin at 0.5, 1, or 2 mM concentrations could preserve significantly higher sperm TM after 4 days; however, only the 1 and 2 mM melatonin concentrations could result in better TM and PM values after 7 days. From day 7 onward, VCL, VAP, VSL, and WOW decreased significantly, but melatonin (1 mM) improved VCL, VSL, and LIN on day 9. In canine sperm, previous studies showed that melatonin supplementation preserved higher acrosome integrity after 7 days of storage, although a significant decrease in the acrosome integrity on day 9 of storage at 5°C was observed without regard to whether melatonin was added (17).

The relationship between the photoperiod and melatonin secretion is well known; in the decreasing photoperiod, the exposure to melatonin increases because melatonin is produced and secreted by night (24). Therefore, seasonally breeding mammals based on photoperiod duration is meant to control their reproductive system based on the daily rhythm of melatonin production. In short-day breeders, this increased melatonin promotes GnRH secretion and regulates gonadotropin and testosterone production (25). Thereby, the seasonal effects on semen quality and biochemical profiles were described in the horse (26), sheep (27), buffalo (28), goat (29), and cattle (30). The protective effects of melatonin on sperm quality can also vary in different seasons, as described in mithun (*Bos frontalis*); in spring, sperm showed higher quality, and the effects of melatonin (decrease in percentages of dead spermatozoa, abnormal spermatozoa, and acrosomal abnormalities) were more intense in spring, followed by autumn and winter (31).

The bitch is considered a non-seasonal female because estrus can occur at any time during the year (32). However, estrus tends to be more frequent in winter and summer (33), even though this fact is supposed to be independent of daylight duration or melatonin concentration (34). On the contrary, sperm is constantly produced throughout the year by males (35). González–Arto et al. (10) studied sperm from donkeys, stallions, boar, bull, dogs, and red deer and suggested that melatonin and melatonin receptors may be universally distributed in the reproductive system of mammals. Given the different dependence of reproduction concerning the photoperiod of these species, melatonin may play a role other than seasonal control and therefore be involved in specific functions on sperm, such as protecting from oxidative damage.

Melatonin levels have shown circadian and diurnal fluctuations in canines (36). Two-fold fluctuations have been described during light-cycle changes in the different seasons (37). The synthesis of melatonin is always highest at night but decreases at dawn, remaining at low levels throughout the day; the lowest levels of this hormone were recorded on summer mornings, whereas the highest concentrations were reached on winter nights (38). The effects of latitude, exercise, and season on melatonin concentration have been described in dogs (39). The peak in melatonin production was more prolonged in high-latitude dogs (65° N) than in lower latitude ones (45° N); at both latitudes, exercise caused a reduction in peak melatonin levels, and melatonin levels were higher in winter than in summer.

In rams (short-day breeder), high levels of melatonin, testosterone, and antioxidant enzymes in seminal plasma have been found during the reproductive season (40). Similar results were obtained when melatonin implantation was carried out during the non-reproductive season (41). These facts suggested a direct action of this pineal hormone on spermatozoa (5). On the contrary, melatonin is present in seminal plasma both during the day and at night (40); the melatonin synthesis by the testes explains the high levels of melatonin in ram seminal plasma during the day (42).

Melatonin supplementation could compensate for the lack of antioxidants in seminal plasma from dogs (21). Melatonin concentration in dog seminal plasma showed a broad range of values due to individual variability, although no effect of age or breed was detected; the potential effect of the season was not analyzed (10). In the literature, there are no data about the seasonal variation of melatonin concentrations in dog seminal plasma. However, if a correlation exists between blood and seminal plasma concentrations of melatonin in dogs, the decreasing photoperiod, related to a higher level of blood melatonin, would cause an increased melatonin level in seminal plasma. As high concentrations of melatonin can impair quality sperm characteristics, the addition of melatonin to sperm in the decreasing photoperiod could create such a high level that it would cause the described negative effects found for acrosomal defects, apoptosis, and viability, which would be opposite to the positive effects achieved in the increasing photoperiod. Unfortunately, no quantification of melatonin in semen samples was performed.

## Conclusion

Concerning chilled dog semen, the negative effects of melatonin were found for acrosomal defects, apoptosis, and viability in the decreasing photoperiod. The addition of melatonin to sperm in the decreasing photoperiod could create such a high level that it would cause the described negative effects. However, the results showed a beneficial effect of melatonin in the increasing photoperiod on acrosomal defects and apoptosis during 0–6 days. Melatonin treatment also increased viability in the short term (days 1 and 2) for both photoperiods. Also, melatonin can provide certain beneficial effects on mitochondrial activity in the medium term (days 2 and 3) in the decreasing photoperiod.

## Data Availability Statement

The raw data supporting the conclusions of this article will be made available by the authors, without undue reservation.

## Ethics Statement

The animal study was reviewed and approved by Committee of Ethics in Animal Experimentation of Universidad de Zaragoza. Written informed consent was obtained from the owners for the participation of their animals in this study.

## Author Contributions

Conceptualization: OM, RA, NM, and MVF. Methodology: NM, JM, MVF, OM, and AMG. Software and formal analysis: MTT. Validation: JM, OM, NM, and MF. Investigation: OM, RA, NM, JM, MTT, AMG, and MVF. Resources, project administration, and Funding acquisition: MVF. Data curation: OM, NM, MTT, and MF. Writing the original draft, writing, reviewing, and editing the manuscript: OM, RA, NM, JM, MTT, AMG, and MVF. Visualization: OM, RA, and NM. Supervision: MTT and MVF. All authors contributed to the article and approved the submitted version.

## Conflict of Interest

RA, NM, and JM were employed by company Magapor S.L. The remaining authors declare that the research was conducted in the absence of any commercial or financial relationships that could be construed as a potential conflict of interest.

## Publisher's Note

All claims expressed in this article are solely those of the authors and do not necessarily represent those of their affiliated organizations, or those of the publisher, the editors and the reviewers. Any product that may be evaluated in this article, or claim that may be made by its manufacturer, is not guaranteed or endorsed by the publisher.
